# The Chloroplast Genome of *Utricularia reniformis* Sheds Light on the Evolution of the *ndh* Gene Complex of Terrestrial Carnivorous Plants from the Lentibulariaceae Family

**DOI:** 10.1371/journal.pone.0165176

**Published:** 2016-10-20

**Authors:** Saura R. Silva, Yani C. A. Diaz, Helen Alves Penha, Daniel G. Pinheiro, Camila C. Fernandes, Vitor F. O. Miranda, Todd P. Michael, Alessandro M. Varani

**Affiliations:** 1 Instituto de Biociências, UNESP - Univ Estadual Paulista, Câmpus Botucatu, São Paulo, Brazil; 2 Departamento de Biologia Aplicada à Agropecuária, Faculdade de Ciências Agrárias e Veterinárias, UNESP - Univ Estadual Paulista, Câmpus Jaboticabal, São Paulo, Brazil; 3 Departamento de Tecnologia, Faculdade de Ciências Agrárias e Veterinárias, UNESP - Univ Estadual Paulista, Câmpus Jaboticabal, São Paulo, Brazil; 4 Ibis Bioscience, Computational Genomics, Carlsbad, California, United States of America; University of Western Sydney, AUSTRALIA

## Abstract

Lentibulariaceae is the richest family of carnivorous plants spanning three genera including *Pinguicula*, *Genlisea*, and *Utricularia*. *Utricularia* is globally distributed, and, unlike *Pinguicula* and *Genlisea*, has both aquatic and terrestrial forms. In this study we present the analysis of the chloroplast (cp) genome of the terrestrial *Utricularia reniformis*. *U*. *reniformis* has a standard cp genome of 139,725bp, encoding a gene repertoire similar to essentially all photosynthetic organisms. However, an exclusive combination of losses and pseudogenization of the plastid NAD(P)H-dehydrogenase (*ndh*) gene complex were observed. Comparisons among aquatic and terrestrial forms of *Pinguicula*, *Genlisea*, and *Utricularia* indicate that, whereas the aquatic forms retained functional copies of the eleven *ndh* genes, these have been lost or truncated in terrestrial forms, suggesting that the *ndh* function may be dispensable in terrestrial Lentibulariaceae. Phylogenetic scenarios of the *ndh* gene loss and recovery among *Pinguicula*, *Genlisea*, and *Utricularia* to the ancestral Lentibulariaceae cladeare proposed. Interestingly, RNAseq analysis evidenced that *U*. *reniformis* cp genes are transcribed, including the truncated *ndh* genes, suggesting that these are not completely inactivated. In addition, potential novel RNA-editing sites were identified in at least six *U*. *reniformis* cp genes, while none were identified in the truncated *ndh* genes. Moreover, phylogenomic analyses support that Lentibulariaceae is monophyletic, belonging to the higher core Lamiales clade, corroborating the hypothesis that the first *Utricularia* lineage emerged in terrestrial habitats and then evolved to epiphytic and aquatic forms. Furthermore, several truncated cp genes were found interspersed with *U*. *reniformis* mitochondrial and nuclear genome scaffolds, indicating that as observed in other smaller plant genomes, such as *Arabidopsis thaliana*, and the related and carnivorous *Genlisea nigrocaulis* and *G*. *hispidula*, the endosymbiotic gene transfer may also shape the *U*. *reniformis* genome in a similar fashion. Overall the comparative analysis of the *U*. *reniformis* cp genome provides new insight into the *ndh* genes and cp genome evolution of carnivorous plants from Lentibulariaceae family.

## Introduction

Carnivorous plants from the genus *Utricularia* (Lentibulariaceae) are distributed worldwide and are comprised of approximately 235 species occurring across every continent except the poles, some arid regions and oceanic islands [1]. They are highly specialized plants with modified leaves (traps) for capturing prey [2,3], and there are diverse life forms, such as aquatic, terrestrial, epiphytic, and reophytic forms [1]. In addition, the genus has some of the smallest nuclear genomes across the angiosperms, ranging from 77 to 561 megabases (Mb; 1C), surpassed only by the genus *Genlisea* with species possessing the smallest genomes [4]. Interestingly, angiosperms present extremes in genome sizes, from around 61 Mb of *Genlisea* to 150,000 Mb of one of the largest plant genomes known, the monocot *Paris japonica* [5,6]. From this perspective, Lentibulariaceae also provides outstanding candidates for model plants, with flexible genomes from around 61 to 1,500 Mb [4,7]; it is thus an important group to address evolutionary, genomic and phylogenetic as well as functional questions. For instance, it is well known that polyploidy, the amount of repetitive DNA such as transposable elements and other repeats, and whole genome duplications (WGD), along with other mechanisms such as small-scale genome duplications or fractionation/gene death rates are the key drivers of genome size differences [6,8,9].

*Utricularia reniformis* A.St.-Hil. is a terrestrial bladderwort endemic to the Brazilian Atlantic Forest and restricted to the mountaintops of the southeastern coast of Brazil [1,10]. All known populations are limited to elevations above 700 m, and live in wet grasslands, wet rocks, and in Bromeliaceae leaf axes [11]. Little is known about the *U*. *reniformis* genome structure. However, previous studies based on microsatellite markers have shown that different populations of *U*. *reniformis* may have tetraploid genomes with high levels of heterozygosity [10]. Recently, the aquatic *U*. *gibba* was sequenced, revealing that its 82 Mb genome encodes a typical number of genes for a plant at 28,500 but that the genome has gone through several whole genome duplications and reductions. In addition, it was reported that the 152kb chloroplast (cp) genome is similar to most other angiosperms [9]. Furthermore, additional studies have focused on genome contraction [12,13] and comparative transcriptomics comparing the different organs and tissues in the aquatic *U*. *gibba* genome [14]. In order to better understand the evolutionary dynamics of a terrestrial form, the *Utricularia reniformis* A.St.-Hil. genome and transcriptome of different tissues are being sequenced and analyzed by our research group.

Four complete cp genomes of the Lentibulariaceae have been published [9,15]. These are *U*. *gibba* and *U*. *macrorhiza*, which are found in aquatic environments as submersed plants, and *Genlisea margaretae* and *Pinguicula ehlersiae*, which are terrestrial species. These four cp genomes are composed of large and small single copy (LSC and SSC) regions and two inverted repeats (IRs), which is the typical circular cp genome quadripartite structure (LSC-IR-SSC-IR). In addition, independent deletions and pseudogenization of subunits of the NAD(P)H dehydrogenase complex (*ndh*), altered proportions of repeats, increase of substitution rates on coding regions and microstructural changes were observed as important landmarks of these cp genomes [15].

The *ndh* complex is composed of eleven genes (*ndhA*, *ndhB*, *ndhC*, *ndhD*, *ndhE*, *ndhF*, *ndhG*, *ndhH*, *ndhI*, *ndhK*, and *ndhJ*), and together with the nuclear genes *nhdL*, *ndhM*, *ndhN*, and *ndhO* encode the thylakoid NAD(P)H dehydrogenase complex. The complex is involved in the electron transfer from NAD(P)H to plastoquinone, protecting the plant cell against photo-oxidative stress, and maintaining optimal rates of cyclic photophosphorylation [16,17]. Interestingly, the *ndh* genes are related to land adaptation and photosynthesis, whereas small changes in any of the *ndh* genes significantly decrease the photosynthesis rate [18]. The *ndh* loss is mainly associated with heterotrophic (parasitic) plants and those endemic to an underwater environment [16]. Indeed, the plants found in submersed aquatic environments receive low levels of light, ultraviolet radiation and have specific limitations that require a number of adaptations, and therefore are under different selective pressures than terrestrial plants [19]. However, the *ndh* genes may be dispensable to the plant. Functional studies suggest that depending on the environmental condition and stress, alternative metabolic pathways might surpass the absence of the *ndh* genes, indicating that these genes may not be central for photosynthesis [19].

However, other questions about the evolution of the Lentibulariaceae cp genomes still remain. For instance, is lateral gene transfer of cp DNA to the nuclear and/or mitochondrial genomes occurring very frequently as it does in other angiosperms [20]. This process is termed endosymbiotic gene transfer [21], and has not been fully addressed in the Lentibulariaceae cp genomes. Furthermore, previous studies suggested that there is a close relationship between carnivorous and parasitic plants at the level of the cp genome [15,22]. Therefore, sequenced cp genomes from this order are still necessary to further understand this relationship, and also to shed light on the systematic relationships in the order Lamiales.

To provide additional insights into the plastid genomes and the driving forces related with plastid NAD(P)H dehydrogenase complex genes loss and pseudogenization in terrestrial carnivorous plants, we have sequenced the cp genome of the *U*. *reniformis*, and compared it to the other cp genomes among other Lamiales, with a specific focus on the carnivorous plants from the family Lentibulariaceae. We have also analyzed the gene content and expression by RNAseq, RNA editing, repeats and structure of the *U*. *reniformis* plastid genome.

## Material and Methods

### Plant Sampling

The *Utricularia reniformis* A.St.-Hil. plant samples were collected in the fall of 2015 near the Serra do Mar Atlantic Forest, located in Salesópolis Municipality, São Paulo State, Brazil (Geographic Location: -23.5047, -45.9018, 961 m a.s.l.) and deposited in the JABU Herbarium of São Paulo State University (voucher V.F.O de Miranda et al., 1670 –JABU). The sample was not collected in protected areas and *U*. *reniformis* is not a threatened species according to the global IUCN (The IUCN Red List of Threatened Species– http://www.iucnredlist.org) and the Brazilian List of Threatened Plant Species [23].

### Chloroplast Sequencing and Assembly

The DNA was extracted following the QIAGEN DNeasy Plant Maxi Kit extraction protocol (QIAGEN). The whole-genome shotgun sequencing was performed using the Illumina MiSeq technology with a paired-end library of 2x300bp and insert size of ~600 bp. The library construction followed the Illumina Nextera XT Preparation Guide (Illumina, USA). The DNA was tagmented (tagged and fragmented) by the Nextera XT transposome. The Nextera XT transposome simultaneously fragments the input DNA and adds adapter sequences to the ends. The tagmented DNA is amplified via a limited-cycle PCR program. The PCR step also adds index 1 (i7) and index 2 (i5) to sequences required for cluster formation. After that, a PCR clean-up was performed by AMPure XP beads to purify the library DNA, and provides a size selection step that removes very short library fragments from the population. A total of 40M paired-end reads were generated and used for the cp genome assembly. Furthermore, in our ongoing studies of the *Utricularia reniformis* genome, we also sequenced 160M mate-pair reads (2x100 bp) with an average of 3,500 bp insert size using the Illumina HiScanSQ technology. The library construction followed the Nextera mate pair gel free protocol (Illumina, USA). The mate-pair set of reads was used in this study to confirm the cp assembly. Poor quality sequences (phred < 24), contaminants, adapters, and sequences with less than 50bp were removed using seqyclean software (https://github.com/ibest/seqyclean), leaving 36M (2x300 bp, paired-end) and 150M (2x100 bp, mate-pair) high quality reads.

The cp genome assembly was conducted in three steps. First, the trimmed reads were mapped back to *Utricularia gibba*, *U*. *macrorhiza*, *Genlisea margaretae*, and *Pinguicula ehlersiae* cp genomes (Accession numbers on S1 Table) with bowtie2 (http://bowtie-bio.sourceforge.net/bowtie2/index.shtml) [24] using default parameters. In the second step, the resulting potential cp reads were then assembled separately with SPAdes v3.7.1 (http://bioinf.spbau.ru/spades) [25], and by iterative (mapping) assembly with MITObim (https://github.com/chrishah/MITObim) [26], using *U*. *gibba*, *U*. *macrorhiza*, *G*. *margaretae* and *P*. *ehlersiae* cp genomes as references. In the third step, the cp genome was manually reconstructed using the SPAdes and MITObim results. The mate-pair reads were mapped back to the cp genome with bowtie2, with the—*very-sensitive* parameter, to confirm the assembly.

### Annotation and Comparative Analysis of the Chloroplast Genomes

The cp genome was annotated using DOGMA (Dual Organellar GenoMe Annotator - http://dogma.ccbb.utexas.edu/)[26] coupled with Prodigal v2.6.2 (http://prodigal.ornl.gov/) [27] and Blast (https://blast.ncbi.nlm.nih.gov) [28] for additional gene location and refinements. The Aragorn software package (http://mbio-serv2.mbioekol.lu.se/ARAGORN/) [29] was used for tRNAs validation and intron identification. Corrections of start and stop codons, and annotation curation was made with Artemis genome browser (http://www.sanger.ac.uk/science/tools/artemis) [30]. In this study the potential pseudogenes were defined by Blast comparative analysis with the use of at least one of the following criteria: (a) presence of at least one stop codon in-frame with the predicted coding region; (b) absence of start and/or stop-codon; (c) frameshift; (d) lacking of at least 20% of the coding region when compared to the respective coding region of related species. For gene assignments the Uniprot (http://www.uniprot.org/) [31] and InterProScan (https://github.com/ebi-pf-team/interproscan) [32] databases were used. The codon and amino acid usage were calculated using CodonW v1.4.4 (http://codonw.sourceforge.net). The circular gene maps were made with OGDRAW (OrganellarGenome DRAW - http://ogdraw.mpimp-golm.mpg.de/) [33]. Comparative analysis was carried out with Interactivenn (http://www.interactivenn.net/) [34], and Blast.

The annotated sequence and the raw reads for the *U*. *reniformis* chloroplast genome have been deposited in the GenBank database under accession number [GenBank: KT336489 and SRR3277235, respectively] (BioProject PRJNA290588).

### Microsatellite and other repeats analysis

Microsatellite analyses were carried out with the MISA software package (http://pgrc.ipk-gatersleben.de/misa/) [35], with thresholds of seven repeats for mononucleotide SSRs, four repeats for di- and trinucleotide SSRs, and three repeats for tetra-, penta- and hexanucleotide SSRs. Maximal number of bases interrupting 2 SSRs in a compound microsatellite was set to 100 bp. Direct and palindromic repeats were determined with REPuter (https://bibiserv2.cebitec.uni-bielefeld.de/reputer) [36], with a minimal size of ≥ 30 bp, sequence identity ≥ 90% (hamming distance of 3).

### Phylogenomic and Phylogenetic Analyses: Maximum likelihood and Bayesian Inference

A total of 47 coding sequences from different chloroplasts were considered (S1A and S1B Table). The alignment of sequences was performed using MAFFT (http://mafft.cbrc.jp/alignment/software/) [37] with default parameters.

The phylogenetic analysis of the plastomes utilized Oleaceae as outgroup. For the probabilistic analysis, the best evolutionary models (best-of-fit) were chosen using ModelTest 3.7 (http://www.molecularevolution.org/software/phylogenetics/modeltest) [38]. Thus, the best-of-fit DNA model was evaluated for each data matrix with the corrected Akaike information criterion [39,40] to estimate the parameters. Maximum likelihood (ML) and Bayesian analyses were performed to estimate the phylogenetic hypothesis for each data matrix. The ML analyses were run with RAxML (http://sco.h-its.org/exelixis/web/software/raxml/index.html) [41]. Prior to the probabilistic analyses, the Akaike information criterion was used to compare the fit to the data of different models as implemented in ModelTest, resulting in the selection of GTR+GAMMA+I as the best-of-fit model. Thus, the GTR+GAMMA+I model was also employed and bootstrapping was applied with 10,000 pseudoreplicates. Bayesian analyses were performed with MrBayes software version 3.2.5 (http://mrbayes.sourceforge.net/) [42] for each data set with 9x10^6^ generations sampled for each 100 generations, using the default parameters. For each analysis, two runs (nruns = 2) with four chains (nchains = 4) were performed beginning from random trees. Initial samples were discarded after reaching stationary (estimated at 25% of the trees). Cladograms (except the one with optimizations of ancestral states) were drawn with the program TreeGraph2 beta version 2.0.52–347 (http://treegraph.bioinfweb.info/) [43].

The phylogenetic analyses of the evolution of *ndh* genes was carried out using a matrix of presence/absence (pseudogenes and frame-shifts were regarded as absences), and the plastome phylogenomic tree (described above) was considered for tracing the presence/absence of *ndh* genes with the Mesquite software version 3.04 (http://mesquiteproject.org). The cloudgram was produced by DensiTree version 2.1 (https://www.cs.auckland.ac.nz/~remco/DensiTree/) [44] based on 18,000 trees sampled with Bayesian inference (using the same parameters as described above for phylogenomic analyses of plastomes) but with the *matK* gene. The *matK* data set was produced by fifty-five sequences from NCBI and also by sequences produced by this study (S1C and S1D Table). The sequences produced in this study were made with 1R-KIM and 3F-KIM primers, following the PCR protocol and procedures recommended by the CBOL Plant Working Group [45].

### RNA-Seq and RNA-edit analyses

Three different plant tissues were used for RNA-seq analysis; fresh leaves, stolons and utricules. The tissues were pooled in three replicates and the total RNA (including the rRNA) was extracted using the PureLink RNA Mini Kit (Thermo Fisher Scientific), according to the manufacturer’s protocol. DNase I (Thermo Fisher Scientific) was used to remove any genomic DNA contamination. The extracted RNA was evaluated using an Agilent 2100 Bioanalyzer (Agilent Technologies) and a Qubit 2.0 Fluorometer (Invitrogen). Only samples having an RNA integrity number (RIN) ≥ 7.0 were used for the sequencing. The cDNA libraries were sequenced on the Ion Proton System generating a yield of 180M of reads with an average read length of 200bp, representing the nuclear and organellar cDNAs. Poor quality sequences (phred < 20), adapters, bacterial contaminants such as photoautotrophic bacteria, and sequences with less than 20bp were removed using prinseq lite v0.20.4 [46]. Two different approaches were used to distinguish potential nuclear/mitochondrial transcripts from authentic plastid transcripts. First, filtered reads were mapped back to the assembled *U*. *reniformis* plastome with bowtie2, with the—*very-sensitive* and—*end-to-end* parameters. The libraries of all three tissues were pooled and this generated a total of 1,632,156 cp related reads with an average length of 170bp for downstream analysis. Second, the RNAseq reads were mapped with CLC Genomics Workbench v9 (QIAGEN Aarhus, Denmark - http://www.clcbio.com) using the following parameters: mismatch cost of 3, insertion cost of 3, deletion cost of 3, minimal alignment coverage of 90% (Length fraction) and similarity fraction of >98%. The cp genes were considered for the RNAseq read mapping and transcription abundance by RPKM (Reads Per Kilobase Million) normalization, whereas only unique read mapping were considered. In addition, the intronic regions of intron-containing genes were also considered for the identification of spliced exons.

The RNA-editing analyses were conducted with TopHat2 (https://ccb.jhu.edu/software/tophat/index.shtml) [47] against the pooled reads using the following parameters: no coverage search (—no-coverage-search), filtering multiple mapped reads such as low complexity or repetitive reads (—prefiltered-multihits), reads spanned by junctions with a minimum of 10 bases (—min-anchor-length 10) or with a maximum of 1 base mismatch in the anchor region (—splice-mismatches 1), to align trans-spliced genes, fusion search was activated (—fusion-search) with the minimum distance of 10,000 (—fusion-min-dist 10,000). The final alignment was inspected for C to U or U to C nucleotide substitutions by a custom perl script, with the use of the following parameters: editing site with a minimum coverage of 10x and phred quality score of ≥25. In addition, the PREP-Cp tool (http://prep.unl.edu/) [48] was used with default parameters to predict additional RNA editing sites.

The cp RNA-seq reads used in this study have been deposited in the GenBank database under accession number [GenBank: SRP072162] (BioProject PRJNA290588).

## Results

### High-quality assembly of the chloroplast genome sequence

A total of 1,259,272 (paired-end, 2x300bp), and 2,087,893 (mate-pair, 2x100bp) chloroplast (cp) related reads were filtered from the raw reads generated by the Illumina MiSeq and HiScanSQ platforms, respectively. These reads shows an average phred value above 30. A total of 1,029,442 (81.7%) paired-reads were assembled to the *U*. *reniformis* cp contigs. This resulted in the assembly of the entire LSC region as well as part of the IRs and the SSC, in three different contigs. The plastid supercontig was manually closed by iterative read mapping and Blast searches. This resulted in a circular sequence with 139,725 bp, with a GC content of 38.15% and average coverage of 3,300x (maximum coverage peak of 8,563x, and standard deviation of 1,725). No high-quality discrepancies were observed, thus indicating a high quality cp genome assembly. A total of 1,751,896 (84%) mate-pair reads were mapped in pairs to the assembled cp genome according to the average insert size of the library (~3,500bp), thus confirming the cp genome assembly. In addition, the mate-pairs read mapping confirmed the assembly of the LSC/IRa, LSC/IRb, and IRa/SSC/IRb boundaries, and therefore the circularity of the plastid supercontig.

Interestingly, the remaining paired-end reads (2x300bp; 229,830; 18.3%) were assembled into contigs containing fragments of incomplete or truncated cp genes (at least 28 contigs encoding 48 truncated cp genes, which span a total of 23,606kbp—S2 Table). Interestingly, these truncated genes are present as full-length gene copies in the cp genome. It is worth noting that the boundaries of some of these contigs do not have similarity to the assembled *U*. *reniformis* cp genome, nor the other Lentibulariaceae cp genomes analyzed. Indeed, during the ongoing genome sequence of the *Utricularia reniformis* A.St.-Hil., we have noted that these cp-derived sequences are interspersed within *U*. *reniformis* mitochondrial (mt) and nuclear genome scaffolds (data not shown). Therefore, this finding suggests that these contigs do not belong to the cp genome sequence itself, and most likely represents distinct endosymbiotic gene transfer events. In addition to these 28 contigs, we have noted an additional number of truncated cp genes interspersed among the *U*. *reniformis* mt genome. For instance, truncated copies of the *ndhJ*, *ndhK* and *ndhC* genes, which are absent in the cp genome (see below), are present in the mt genome. These findings strongly support that gene transfer events between the organelles and nuclear DNA occurred and may shaped the *U*. *reniformis* genome.

### Organization and gene content of the *U*. *reniformis* plastid genome

**Fig 1:**
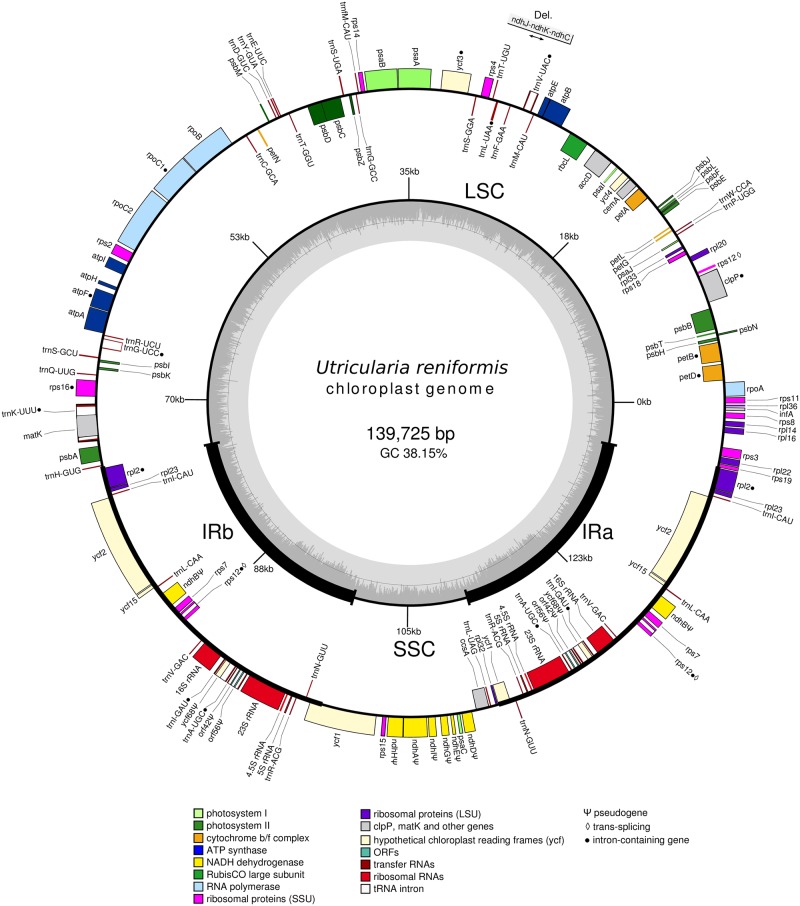
Genomic map of the *Utricularia reniformis* cp genome. Genes shown on the outside of the map are transcribed clockwise, whereas genes on the inside are transcribed counter-clockwise. Genes are color coded by their function in the legend.

*Utricularia reniformis* circular cp genome size is 139,725 bp in length, showing the typical quadripartite structure found in most land plants. The IRs spans 24,064bp each (34%), whereas the small single copy (SSC), and large-single-copy region (LSC) span 12,661bp (10%) and 78,936bp (56%), respectively (Fig 1). The *ndhJ*, *ndhK* and *ndhC*, which are commonly located on the LSC in angiosperms, are lost in the *U*. *reniformis* cp genome (Fig 1). However, with that exception, the *U*. *reniformis* cp LSC is collinear in gene content and arrangement to the respective region of the closely related species, *U*. *gibba*, *U*. *macrorhiza*, *G*. *margaretae* and *P*. *ehlersiae* and to other angiosperms, such as *Arabidopsis thaliana* and *Nicotiana tabacum*. The GC content of the LSC and SSC regions are 36% and 31.75%, respectively, whereas that of the IR regions is 43%. As observed in other land plants, such as from *Cynara humilis*, a species of family Asteraceae, the higher GC content in the IRs is due to the GC rich rRNA genes *rrn16*, *rrn23*, *rrn4*.5, and *rrn5* [49].

There are a total of 136 predicted coding regions, 90 of which are single copy (68 CDS and 22 tRNAs), and 46 of which are duplicated in the IRs (22 CDS, 16 tRNAs and 8 rRNAs) (Table 1). In addition to the predicted coding regions, 14 pseudogenes, 6 of which are single copies, mostly located on the SSC and related with *ndh* complex genes, were identified (Fig 1 and Table 1). A total of 22,601 codons represent the coding repertoire of the protein coding regions (Table 2). The most prevalent codon encodes for leucine (2,326–5.99%), whereas the least is cysteine (243–1.07%). Only four coding regions have alternative start codons, these are the *rpl16* (AUC), *rps19* (GUG) and *ycf15* (GUG). All 3 of the stop codons are present, with UAA being the most frequently used (UAA 51%, UAG 27% and UGA 21%). The predicted tRNA genes enable the *U*. *reniformis* cp genome to decode all amino acids, but not all 61 codons (29 out 64 codons; Table 2). A similar codon distribution is also observed with the related species *U*. *gibba*, *U*. *macrorhiza*, *G*. *margaretae* and *P*. *ehlersiae*.

**Table 1:** List of genes encoded by the *Utricularia reniformis* chloroplast (cp) genome.

**Photosystem I**	*psaA*, *psaB*, *psaC*, *psaI*, *psaJ*
**Protosystem II**	*psbA*, *psbB*, *psbC*, *psbD*, *psbE*, *psbF*, *psbH*, *psbI*, *psbJ*, *psbK*, *psbL*, *psbM*, *psbN*, *psbT*, *psbZ*
**Cytochrome b/f complex**	*petA*, *petB*•, *petD*•, *petG*, *petL*, *petN*
**ATP synthase**	*atpA*, *atpB*, *atpE*, *atpF*•, *atpH*, *atpI*
**NADH dehydrogenase**	*ndhA* Ψ, *ndhB* Ψ (2x), *ndhD* Ψ, *ndhE* Ψ, *ndhG* Ψ, *ndhH* Ψ, *ndhI* Ψ
**RubisCO large subunit**	*rbcL*
**RNA polymerase**	*rpoA*, *rpoB*, *rpoC1*•, *rpoC2*
**Ribosomal proteins (SSU)**	*rps2*, *rps3*, *rps4*, *rps7* (2x), *rps8*, *rps11*, *rps12* ◊•(2x), *rps14*, *rps15*, *rps16* •, *rps18*, *rps19*
**Ribosomal proteins (LSU)**	*rpl2* (2x)•, *rpl14*, *rpl16*, *rpl20*, *rpl22*, *rpl23* (2x), *rpl32*, *rpl33*, *rpl36*
**Other genes**	*ccsA*, *clpP* •, *matK*, *accD*, *cemA*, *infA*
**hypothetical chloroplast reading frames**	*ycf1* (2x), *ycf2* (2x), *ycf3* •, *ycf4*, *ycf15* (2x), *ycf68* Ψ(2x), *orf42* Ψ(2x), *orf56* Ψ(2x)
**Transfer RNAs**	*trnA*-UGC (2x)•, *trnC*-GCA, *trnD*-GUC, *trnE*-UUC, *trnF*-GAA, *trnG*-GCC, *trnG*-UCC•, *trnH*-GUG, *trnI*-CAU (2x), *trnI*-GAU (2x)•, *trnK*-UUU•, *trnL*-CAA (2x), *trnL*-UAA•, *trnL*-UAG, *trnM*-CAU, *trnN*-GUU (2x), *trnP*-UGG, *trnQ*-UUG, *trnR*-ACG (2x), *trnR*-UCU, *trnS*-GCU, *trnS*-GGA, *trnS*-UGA, *trnT*-GGU, *trnT*-UGU, *trnV*-GAC (2x), *trnV*-UAC•, *trnW*-CCA, *trnY*-GUA, *trnfM*-CAU
**Ribosomal RNAs**	*rrn4*.*5* (2x), *rrn5* (2x), *rrn16* (2x), *rrn23* (2x)

^Ψ^ pseudogene

^◊^ trans-splicing

^•^ intron-containing gene

**Table 2:** Codon usage table. Codon usage and codon—anticodon recognition pattern for tRNA in *Utricularia reniformis* cp genome.

Amino acid	Codon	No.	RSCU (a)	%(b)	tRNA	Amino acid	Codon	No.	RSCU (a)	%(b)	tRNA
Ala	GCU	479	1.64	40.9	-	Pro	CCU	339	1.42	35.6	-
	GCC	201	0.69	17.2	-		CCC	214	0.90	22.4	-
	GCA	346	1.18	29.6	trnA-UGC •		CCA	240	1.01	25.2	trnP-UGG
	GCG	143	0.49	12.3	-		CCG	159	0.67	16.8	-
Cys	UGU	181	1.49	74.4	-	Gln	CAA	629	1.51	75.3	trnQ-UUG
	UGC	62	0.51	25.6	trnC-GCA		CAG	206	0.49	24.7	-
Asp	GAU	702	1.60	79.9	-	Arg	CGU	301	1.24	20.7	trnR-ACG
	GAC	176	0.40	20.1	trnD-GUC		CGC	115	0.47	7.9	-
Glu	GAA	878	1.50	74.9	trnE-UUC		CGA	338	1.39	23.3	-
	GAG	294	0.50	25.1	-		CGG	128	0.53	8.7	-
Phe	UUU	806	1.31	65.4	-		AGA	407	1.68	27.9	trnR-UCU
	UUC	426	0.69	35.6	trnF-GAA		AGG	168	0.69	11.5	-
Gly	GGU	483	1.27	31.7	-	Ser	UCU	475	1.67	27.8	-
	GGC	184	0.48	12.2	trnG-GCC		UCC	275	0.97	16.1	trnS-GGA
	GGA	555	1.46	36.4	trnG-UCC •		UCA	320	1.12	18.7	trnS-UGA
	GGG	300	0.79	19.7	-		UCG	198	0.70	11.6	-
His	CAU	420	1.49	74.6	-		AGU	345	1.21	20.2	-
	CAC	142	0.51	25.4	trnH-GUG		AGC	95	0.33	5.6	trnS-GCU
Ile	AUU	916	1.50	49.9	-	Thr	ACU	464	1.54	38.3	-
	AUC	369	0.60	20.1	trnI-GAU		ACC	234	0.77	19.4	trnT-GGU
	AUA	548	0.90	30	trnI-CAU •		ACA	366	1.21	30.3	trnT-UGU
Lys	AAA	990	1.48	73.9	trnK-UUU •		ACG	144	0.48	12	-
	AAG	348	0.52	26.1	-	Val	GUU	457	1.46	36.5	-
Leu	UUA	704	1.82	30.3	trnL-UAA •		GUC	163	0.52	13.1	trnV-GAC
	UUG	493	1.27	21.2	trnL-CAA		GUA	466	1.49	37.2	trnV-UAC •
	CUU	501	1.29	21.5	-		GUG	166	0.53	13.2	-
	CUC	148	0.38	6.3	-	Tyr	UAU	642	1.52	76	-
	CUA	312	0.80	13.4	trnL-UAG		UAC	202	0.48	24	trnY-GUA
	CUG	168	0.43	7.3	-	Trp	UGG	384	1.00	100	trnW-CCA
Met (START)	AUG	480	1.00	100	trnf(f)M-CAU	Stop	UGA	16	0.63	21	-
Asn	AAU	843	1.49	74.6	-		UAA	39	1.54	51	-
	AAC	287	0.51	25.4	trnN-GUU		UAG	21	0.83	27	-

^(a)^ Relative Synonymous Codon Usage

^(b)^ Codon frequency (in %) per amino acid

^•^Intron-containing tRNA genes

Other features are related with the genes *atpF*, *rpl2*, *rps16*, *rpoC1*, *rps16*, *petB* and *petD* which each contain one intron, and *clpP* and *ycf3* which each contain two introns. The introns of all protein-coding genes share the same splicing mechanism as Group II introns. The *rps12* gene is transpliced, the 5’ end exon is located in the LSC region and the 3’ exon and intron are duplicated and located in the IR regions. This is frequently observed in other angiosperms as well [50,51]. The splicing was also evidenced by mapping of the RNA-seq reads on spliced exons including those from transpliced genes *rps12*. Conversely, six tRNAs contain introns. In the IRs, the *trnI*-GAU intron includes the *ycf68* pseudogene, and *trn*A-UGC intron includes the *orf42* and *orf56* pseudogenes. On the LSC, *trn*G-UCC, *trn*V-UAC and *trn*L-UAA each contain one intron, and the *trn*K-UUU intron includes the *matK* gene. The *matK* encodes for a maturase which is not able to promote intron mobility due to the absence of the reverse transcriptase domain, and the *trnL*-UAA contains a group I intron. This genomic organization is highly conserved in the *U*. *gibba*, *U*. *macrorhiza*, *G*. *margaretae* and *P*. *ehlersiae*.

In summary, 68,969 bp (49.4%) of the cp genome is made up of coding DNA, whereas 57,159 bp (40.9%) 2,766 bp (2%), and 9,044 bp (6.4%) corresponds to CDS, tRNA and rRNAs, respectively. Introns represent 16,811 bp (12%), pseudogenes 6,453 bp (4.6%), and the remaining 47,492 bp (34%) of the genome is made up of non-coding and intergenic spacers.

### Transcription evidence of the plastidial genes

Due to its endosymbiotic origin, the chloroplast retains a prokaryotic biochemistry, with its own gene expression machinery. However, the expression of cp genes in many land plants requires different nuclear-encoded proteins, mostly to bind transcripts and regulate translation [52]. Conversely, transcription of some genes is regulated by light-dark cycles or nutrient availability, such as those from photosystem I and II, and *rbcL*, and the results presented here show that *U*. *reniformis* follows this trend. Moreover, the chloroplast RNAs shows a poly(A) tail, which may target the cp RNAs for rapid decay [53]. In order to explore the *U*. *reniformis* cp genome expression, cDNA libraries from three different tissues (leaves, stolon, and utricules) were created and sequenced (RNAseq).

**Fig 2:**
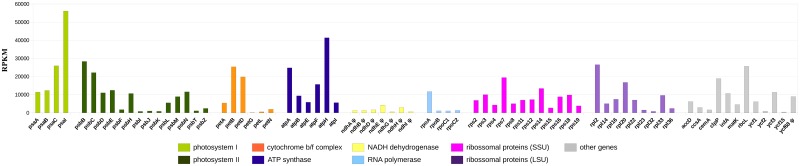
Expression profile of the *Utricularia reniformis* cp genes. The expression values are normalized in Reads Per Kilobase per Million mapped reads (RPKM) on the Y-axis. The *psaJ* and *psbA* genes show the highest expression levels as measured in RPKM (323,750 and 437,421, respectively) and were suppressed from the figure for clarity.

The libraries were merged, and a total of 113,224 (7%, out from 1,632,156) reads were uniquely mapped to the annotated cp genes, and the expression profiles of the cp genes were investigated. Overall, >95% of the coding regions and pseudogenes have at least one read of coverage (average base coverage of 183x and median of 33x). The remaining reads (1,087,926; 65.5%) map to the cp rRNAs, such as the 23S rRNA, and also to the tRNAs, and 431,006 (27.5%) reads are non-specific matches. These results show that the cp genome is transcriptionally active, and that all predicted genes, including the pseudogenes are transcribed at some level (Fig 2 and S3 Table). The transcription of genes from photosystem I and II are highly expressed compared to the other genes. In particular the *psaJ* and *psbA* genes show the highest expression levels as measured in RPKM (323,421 and 437,421, respectively; S3 Table), and were removed from Fig 2 for brevity and clarity. Moreover, the genes *psaC*, *psaI*, *psbB*, *psbC*, *petB*, *atpA*, *atpH*, *rpl2*, *clpP*, and *rbcL* show expression near or above 20,000. The least expressed gene is the *petG*, which encodes the cytochrome b6/f complex subunit V,with expression value of 77, and only one RNAseq read mapped (covering >95% of *petG* with 99% of identity). Other genes with low RPKM expression values are: *psbF* (15 transcripts), *psbI* (11 transcripts), *psbJ* (12 transcripts), *psbK* (7 transcripts), *psbT* (6 transcripts), *petL* (2 transcripts), *rpoB* (409 transcripts), *rpoC1* (246 transcripts), *rpoC2* (566 transcripts), *rpl23* (11 transcripts), *rpl32* (5 transcripts), *ycf15* (11 transcripts) and *ycf2* (575 transcripts)(More details in S3 Table).

Another remarkable feature observed is related to the transcriptional activity of pseudogenes. Interestingly, all *ndh* complex genes have some sort of mutation, which lead to stop codons in-frame, frameshift and deletions (detailed analysis in the next sections). However, RNAseq reads were mapped to each of the *ndh* genes (≥98% identity considering 100% of the read length), thus suggesting that these pseudogenes are transcribed. Moreover the *ycf68*, *orf42* and *orf56* pseudogenes also show expression values in RPKM. The *ycf68* gene has a frameshift and one stop codon in-frame, whereas *orf42* has one stop codon in-frame, and *orf56* has a frameshift. The *ycf68* gene encodes a functional protein present in grasses, gymnosperm and Nymphaeales, whereas *orf42* and *orf56* are commonly found in the chloroplast genomes of the other species [54,55]. However, *orf42* and *orf56* are related to mitochondrial genes [55], showing a considerable sequence similarity, and therefore the expression results observed in both genes should not be fully considered due to non-specific and misleading alignments, and therefore they are not shown in Fig 2. Comparative analysis did not detect copies of the *ndh*, *orf42*, *orf56* and *ycf68* genes in nuclear or mitochondrial scaffolds, suggesting that these are really lost from *U*. *reniformis*, and thus indicating that these represent real transcripts from a pseudogene template. Interestingly, previous studies indicate that truncated, transcribed molecules may exist in the chloroplast [56], and may support these findings. However, whether these transcripts encode stable RNAs, or even if they are translated to functional proteins is yet to be established, and their roles remain unknown.

Overall, these results indicate that essential cp genes are expressed. However, whether these pseudogenes are not completely inactivated yetor if they encoding regulatory RNAs, orifthe photosystem is impaired or not, due to the loss and pseudogenization of *ndh* complex, and if some sort of *ndh* functional replacement could or is occurring remains unknown.

### Identification and prediction of RNA editing sites

RNA editing is a post-transcriptional modification that normally changes a cytosine (C) to a uracil (U) or U to C nucleotides, producing transcripts that are different from their DNA template [57]. These modifications can alter the amino-acid sequence of protein, and can also introduce new start and/or stop codons [57]. At least 44 potential editing sites were identified using PREP-Cp and RNAseq analysis. These are all in the coding region, whereas six editing sites were exclusively detected by RNAseq data corresponding to potential novel editing sites (Tables 3 and 4). The editing level from RNAseq data was inferred from the C versus U ratio of the transcripts derived from the respective loci. Among the RNAseq detected sites, three editing sites alters the first and the second nucleotide of a codon, and one editing site alters the third nucleotide of a codon. Except for *rpoA*, all editing sites lead to non-synonymous substitutions. Four sites located in *rpoB*, *rps14*, *petB* and *rps2* are the most frequent editing sites (> 81%), whereas the remaining sites located in *atpA*, *psaB* and *rpoA* were edited at low frequency (11 to 20%) (Table 3). Moreover, the PREP-Cp predicted 37 additional sites, whereas one site located on the *rpoB* gene was confirmed by RNAseq data (Table 3). All predicted sites by PREP-Cp lead to non-synonymous substitutions. Overall, except for the six editing site detected by RNAseq, the RNA editing sites are quite similar to those observed in other angiosperms [58]. Therefore, these results suggest that the prediction tools may fail to identify authentic RNA editing sites, and that RNAseq data can be used preferentially.

**Table 3:** RNA editing pattern in *Utricularia reniformis* cp genome identified by RNAseq reads alignment.

Gene	Genome (cp)		Codon		Codon position	Amino acid		Editing level
	Position	Strand	from	to		from	to	U/C (%)
*atpA*	129,471	+	CAC	UAC	1	H	Y	15
*psaB*	102,612	-	CCU	UCU	1	P	S	20
*petB*	68,312	-	CCA	UCA	1	P	S	99
*rpoA* *	65,985	+	AAC	AAU	3	N	N	11
*rpoB* †	115,658	+	UCU	UUU	2	S	F	81
*rps2*	124,300	+	ACA	AUA	2	T	I	99
*rps14*	103,606	+	CCA	UCA	2	P	S	85

* synonymous substitution

^†^ Also predicted by PREP-Cp

**Table 4:** RNA editing pattern in *Utricularia reniformis* cp genome predicted by PREP-Cp.

Gene	Genome (cp)		Codon		Codon position	Amino acid		Editing Score
	Position	Strand	from	to		from	to	(PREP-Cp)
*accD*	86,311	-	ACG	AUG	2	T	M	1.0
	86,391	-	CGG	UGG	1	R	W	1.0
	86,562	-	CCU	UCU	1	P	S	1.0
	87,043	-	UCG	UUG	2	S	L	0.8
*atpI*	125,155	+	UUT	CUU	1	P	L	1.0
	125,752	+	UCA	UUA	2	S	L	1.0
*ccsA*	35,170	-	GCC	GUV	2	A	V	1.0
*matK*	136,847	+	UCT	UAU	3	H	Y	1.0
	137,456	+	CAU	UAU	1	H	Y	1.0
	137,579	+	CCG	UCG	1	P	S	0.86
*petB*	68,692	-	CGG	UGG	1	R	W	1.0
	68,885	-	CCA	CUA	2	P	L	1.0
*psaI*	84,202	-	CCU	UCU	1	P	S	1.0
*psbB*	70,971	-	CGU	UGU	1	R	C	1.0
	71,044	-	GCG	GUG	2	A	V	0.86
*rpl2* ‡	1,364 60,409	- +	GCG	GUG	2	A	V	0.86
*rpl20*	75,977	+	UCA	UUA	2	S	L	0.86
*rpoA*	65,590	+	UCA	UUA	2	S	L	1.0
*rpoB*	113,701	+	CUU	UUU	1	L	F	1.0
	113,996	+	UCU	UUU	2	S	F	1.0
	114,131	+	UCA	UUA	2	S	L	0.86
	114,209	+	UCA	UUA	2	S	L	1.0
	114,575	+	ACG	AUG	2	T	M	0.86
	115,219	+	CUC	UUC	1	L	F	1.0
†	115,657	+	UCU	UUU	2	S	F	1.0
	116,084	+	UCA	UUA	2	S	L	0.86
*rpoC1*	119,318	+	CGU	UUA	1	R	C	0.86
*rpoC2*	120,709	+	CUU	UUU	1	L	F	0.86
	121,870	+	CUU	UUU	1	L	F	1.0
	122,038	+	CUU	UUU	1	L	F	1.0
	122,170	+	CGG	UGG	1	R	W	1.0
	123,590	+	UCA	UUA	2	S	L	0.86
*rps14*	103,537	+	UCA	UUA	2	S	L	1.0
	103,606	+	CCA	CUA	2	P	L	1.0
*rps16*	134,633	+	UCA	UUA	2	S	L	1.0
*rps2*	124,189	+	ACC	AUC	2	T	I	1.0
	124,414	+	UCA	UUA	2	S	L	1.0

^‡^ The *rpl2* is duplicated in the IRs

^†^ Also detected by RNAseq read mapping

Several plastids transcripts require C to U editing, whereas *ndh* genes contain about 50% of the editing sites of angiosperm plastid transcripts [18]. However, the RNA edition was also observed in pseudogenes, such as the *ndhB* [58]. Interestingly none of the editing sites were associated with transcripts that align with *ndh* genes loci, supporting the idea that despite the fact that RNAseq reads align to these genes they may be indeed correspond to non-functional genes.

### Microsatellite and other repeats

**Fig 3:**
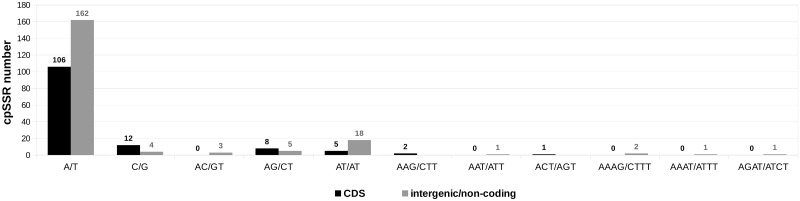
Frequency of SSR motifs found in *Utricularia reniformis* cp coding and intergenic regions, taking into account sequence complementarities.

It was previously described that Lentibulariaceae plastomes carry a large number of chloroplast microsatellite (cpSSR) and small number of repeats longer than 60 bp [15]. The *U*. *reniformis* plastome follows this trend, having at least 331 cpSSR ranging from 7 to 179 bp (Fig 3). Among those, mono and di repeats were the most common, representing 86% (284 cpSSRs) and 4% (13 cpSSRs), respectively. No pentanucleotides or hexanucleotides repeats were found, and low frequencies of tri-, and tetra repeats were observed (Fig 3). Among the 284 mononucleotide repeats, only 16 C/G type repeats were found, with all other repeats belonging to the A/T type. Repeat number of mononucleotide motifs ranged from seven (48%) to 15. Furthermore, at least 55 cpSSRs mononucleotide repeats with a length of at least 10 bp were detected. It is noteworthy that the AT-rich di repeats are commonly found in others carnivorous plant plastomes [15]. In general, these results are also quite similar to those observed in *Tanaecium tetragonolobum*, from the family Bignoniaceae, where 347 cpSSR were identified [59]. The distribution of the mononucleotide, di-repeats, tri-repeat and compound/polymorphic cpSSRs are shown in Table 5, and indicate a similar distribution of cpSSRs to that present in the coding regions of *U*. *gibba*, *U*. *macrorhiza*, *G*. *margaretae* and *P*. *ehlersiae*. However, the majority of the cpSSR are located in non-coding/intergenic regions, accounting for up to 197 (60%) occurrences, whereas 38 were polymorphic, representing good regions for the development of cpSSRs molecular markers for population studies and to estimate the relationship between different Lentibulariaceae.

**Table 5:** Distribution of the cpSSRs present in the *U*. *reniformis* cp coding regions.

cpSSR type	Genes	Pseudogenes
mono-repeats	*accD*, *atpB*, *atpF*, *ccsA*, *clpP*, *matK*, *petA*,*psaA*, *psaI*, *psbB*, *psbC*, *psbF*, *psbK*, *psbT*, *rpl14*, *rpl20*, *rpl2*, *rpl22*, *rpoA*, *rpoB*, *rpoC1*, *rpoC2*, *rps11*, *rps3*, *rps8*, *ycf1*, and *ycf2*	*ycf68*, *orf56*, *ndhA*, *ndhB*, *ndhD*, *ndhI* and *ndhG*
di-repeats	*petA* (AT), *psaA* (TC), *rpoB* (TA), *rpoC2* (AT), and *ycf2* (TA)	*ndhB* (AG)
tri-repeat (TTC)	*psbC*	
compound/polymorphic	*ccsA*, *psaA*, *rpl32*, *rpoC2*, *rps4*, *rps7*, *rps14*, *rps15*, *rps18*, *rps19*, *ycf1*, and *ycf2*	*ndhA* and *ndhI*

A plethora of forward and palindromic repeats were also identified in the *U*. *reniformis* cp genome (Table 6). A total of 24 pairs of repeats (30 bp or longer, and up to 58 bp) were identified. These repeats are spread out over the LSC (42%), IRs (50%) and SSC (8%), and no introns were found to contain repeated elements. These repeats are found predominantly in coding regions (58%), which are not commonly found in other angiosperm lineages [51], but are frequent in other Lentibulariaceae [15]. This may indicate that the Lentibulariaceae cp coding regions are repeat hotspots acting as a source of recombination and rearrangements. For instance, two palindromic repeats were identified within the genes *psaC*, *ndhD*Ψ and *ccsA*, suggesting a potential role during the pseudogenization process of *ndhD*. In addition, the region containing *psaC*, *ndhD* and *ccsA* in *U*. *reniformis*, *U*. *gibba*, *U*. *macrorhiza*, *P*. *ehlersiae* and *G*. *margaretae* is quite variable in terms of nucleotide and gene composition, and order (see below). Palindromic repeats located on the LSC are identified within the genes *accD*, *rbcL*, *ycf3*, *psaA*, *psaJ*, *rpl2*, *rpl33*, *rps14*, *rps19* and *psaB*, and the majority of repeats located on the IRs are associated with the gene *ycf2*. In summary, the number and distribution of these sequences vary from one species to another. However, comparative analyses indicate that this repeat repertoire is quite similar to those previously observed in the terrestrial forms *G*. *margaretae* and *P*. *ehlersieae*.

**Table 6:** Sequence repeats in the cp genome of *Utricularia reniformis*. Type, location and size (in bp) of repeated elements (IGS, Intergenic spacer).

Type	Location	Size (bp)
Palindrome	IGS: *ndhD*Ψ-*ccsA*	58
Palindrome	IGS: *accD*-*rbcL*	44
Palindrome	*rps19* | IGS:*trnH*-GUG-*rpl2*	37
Palindrome	IGS: *ycf3*-*psaA*	36
Palindrome	*trnS*-GGA-*trnS*-GCU	30
Forward	*psaA*-*psaB*	35
Forward	IGS: *rps12*_end–*trnV*-GAC	31
Palindrome	IGS: *rps12*_end–*trnV*-GAC | IGS: *trnV*-GAC-*rps7*	31
Palindrome	IGS: *rps1*2_end–*trnV*-GAC | IGS: *trnV*-GAC-*rps7*	31
Forward	IGS: *trnV*-GAC-*rps7*	31
Palindrome	IGS: *rpl33*-*psaJ*	33
Palindrome	IGS: *rps14* –*trn(f)M*-CAU	30
Forward	*trnS*-UGA-*trnS*-GCU	32
Forward	*ycf2*	31
Palindrome	*ycf2-ycf2*	31
Palindrome	*ycf2-ycf2*	31
Forward	*ycf2*	31
Forward	*ycf2*	30
Palindrome	*ycf2-ycf2*	30
Palindrome	*ycf2-ycf2*	30
Palindrome	IGS: *psaC*-*ndhD*Ψ	30
Forward	*ycf2*	30
Palindrome	*trnS*-GGA | IGS: *trnS*-UGA–*psbC*	30
Forward	*trnG*-GCC-*trnG*-UCC	30

### Comparative analysis among other Lentibulariaceae

#### Terrestrial and aquatic forms show distinct SSC organization

**Fig 4:**
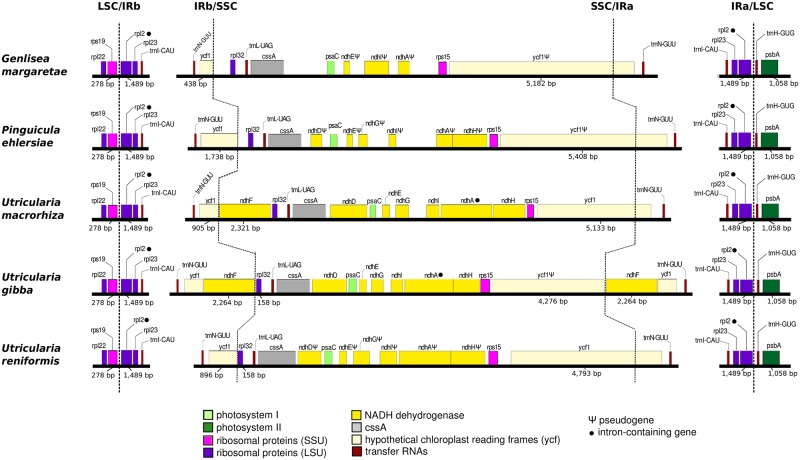
Comparison of the boundaries of the LSC, SSC, and IR regions in the currently available cp genomes of Lentibulariaceae.

The plastomes of the sequenced Lentibulariaceae are highly conserved in terms of gene synteny. Fig 4 shows comparisons of the SSC region and the IR-LSC and IR-SSC boundaries. In spite of the high level of synteny noted on the LSC/IRb and IRa/LSC junctions, the IRb/SSC, SSC/IRa boundaries and the SSC region itself show distinct organization. Gene deletions, rearrangements, expansions and contractions in the SSC and IR/SSC boundaries of these plants are markedly noted.In the SSC, only the *rpl32*, *trnL*, *cssA*, *psaC* and *rpl15* genes are conserved among the analyzed plants. Moreover, a distinct repertoire for the *ndh* gene complex is observed in each plastome. Indeed, previous studies have indicated that several mutational hotspots were found in the entire SSC as well as the region around the *ndh* genes of *U*. *macrorhiza*, *G*. *margaretae* and *P*. *ehlersiae* [15]. This may be one of the evolutionary mechanisms related to the *ndh* and *ycf1* pseudogenization process.

The *ndhF* gene is exclusively present in aquatic species, and only *U*. *gibba* carries two copies of the *ndhF* gene, delimiting the IR/SSC boundaries. It is worth noting that the *ndhF* genes, which are commonly located on the IR/SSC boundaries in other angiosperms plastomes, are lost in the other Lentibulariaceae terrestrial taxa. The loss of the *ndhF* is not an exclusive feature of the terrestrial forms, since this gene can be either present or absent in angiosperms [51,60]. However *ndhF* is often found in Coniferophyta, Filicophyta, Ginkgophyta, Gnetophyta, Lycophyta, Psilophyta and Sphenophyta plastomes [61,62]. Interestingly, the *ndhF* loss may be related to shifts in the position of the junction of the IR and SSC regions in Orchidaceae [62]. Indeed these shifts may lead to losses due to recombination, as observed in the Lentibulariaceae (Fig 4).

For all the other species, two copies of the *ycf1* gene, one larger and other smaller, delimits the IR/SSC boundaries. Interestingly, assuming that there have been no major errors in genome assembly, the *ycf1* gene shows different sizes among the analyzed plants, indicating that together with *ndhF*, these genes may be used as a potential hotspot for the study of the evolution of the IR/SSC junction in the Lentibulariaceae. For instance, in *G*. *margaretae*, *P*. *ehlersiae* and *U*. *gibba*, the larger copy of *ycf1* is a pseudogene, whereas all smaller copies are intact genes. In addition, *ycf1* is often associated with many rearrangements in other angiosperms [63], and this is indeed observed within the Lentibulariaceae plastomes. Therefore, as observed in other land plants, during the course of evolution, the Lentibulariaceae plastomes displayed rearrangements, deletions and gene loss. Overall, these finding also suggests a correlation of the plant life style with plastome genomic structure. For instance, whereas aquatic forms of Lentibulariaceae tend to maintain larger SSC regions, retaining the *ndh* complex genes intact, the terrestrial forms have smaller SSC regions and have lost *ndh* genes.

#### The Lentibulariaceae plastome gene repertoire varies mainly in the *ndh* genes

**Fig 5:**
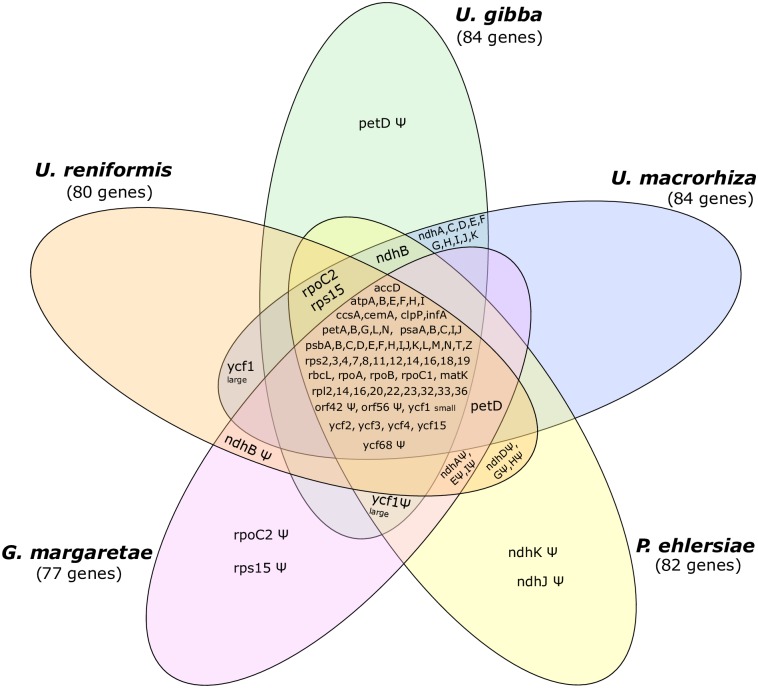
Venn diagram showing the full complement of genes present in the sequenced Lentibulariaceae cp genomes (pan genome). The tRNAs and rRNAs are not included. The numbers below each species represent the unique genes used in the comparison.

The gene repertoires of the plastome of the sequenced carnivorous plants (*U*. *gibba*, *U*. *macrorhiza*, *G*. *margaretae*, and *P*. *ehlersiae*) are quite similar (Fig 5). The Lentibulariaceae cp core gene repertoire is composed of 69 genes, mostly involved in photosynthesis, energy metabolism, and other housekeeping functions (Fig 5, center). Indeed, this result is similar to the core cp genome of essentially all photosynthetic organisms [52]. The main differences are related to a combination of losses and pseudogenization of *ndh* genes among the five plastomes. It is worth noting that the *ndh* gene loss/pseudogenization observed in terrestrial forms are clearly derived from independent events (Table 7), which is corroborated by previous studies [15]. In *Genlisea*, the genes *ndhC*, *D*, *F*, *G*, *H*, *J*, and *K* were lost from the plastome, and the genes *ndhA*, *B*, *E*, and *I* are truncated, whereas the *ndhA*, *D*, *E*, *G*, *H*, *I*, *J*, and *K* retain insertion/deletions and frame shifts in *Pinguicula*[15]. However, *U*. *reniformis* shows a different pattern, in that the genes *ndhC*, *F*, *J* and *K* were lost from the plastome, and the genes *ndhA*, *B*, *DE*, *G*, *H* and *I* reside as truncated pseudogenes. Previous studies indicate that the *ndh* gene loss in *Genlisea* and *Pinguicula* occurred two times independently within Lentibulariaceae [15]. Interestingly, considering only carnivorous plants, the *ndh* gene complex spans to up to 16kb in aquatic forms, representing about of 10% of the plastome, whereas terrestrial forms vary from 5kb to 10kb (3–7%—Table 7).

**Table 7:** Distribution and comparisons of the eleven genes in the *ndh* complex, encoded in aquatic and terrestrial cp genomes from carnivorous plants and the ancestral lineages *Sesamum indicum*, *Tanaecium tetragonolobum* and *Andrographis paniculata*. Black box represents that the given *ndh* gene is present and intact. Gray box indicates truncated *ndh* gene according to the legend. White box indicate that the given *ndh* gene is absent in the cp genome.

	*Sesamum indicum* (terrestrial)			*Tanaecium tetragonolobum* (terrestrial)			*Andrographispaniculata*(terrestrial)			*Pinguicula ehlersiae* (terrestrial)			*Genlisea margaretae* (terrestrial)			*Utricularia reniformis* (terrestrial)			*Utricularia macrorhiza* (aquatic)			*Utricularia gibba* (aquatic)		
***ndhA*** • **SSC**																								
																							
	2,171bp			2,162bp			2,069bp			1,811bp ■ □▲			197bp ▲			1,640bp ■ □			2,190bp			2,153bp		
***ndhB***• * IR																								
																							
	2,211bp			2,211bp			2,21bp			2.211bp			2,121bp ■ □			1,080bp ■ ▲			2,211bp			2,211bp		
***ndhC*** **LSC**																								
																							
	362bp			362bp			362bp												362bp			362bp		
***ndhD*** **SSC**																								
																							
	1,502bp			1,148bp ▲			1,454bp			622bp □ ▲						737bp ■ ▲			1,526bp			1,526bp		
***ndhE*** **SSC**																								
																							
	305bp			305bp			305bp			309bp □			188bp ■ ▲			233bp ■ ▲			305bp			305bp		
***ndhF*** **SSC**																								
																							
	2,255bp			2,231bp			2,23bp												2,261bp			2,261bp (*)		
***ndhG*** **SSC**																								
																							
	530bp			530bp			530bp			520bp □						509bp ■			530bp			530bp		
***ndhH*** **SSC**																								
																							
	1,18bp			1,181bp			1,18bp			1,131bp □						1,085bp ■ ▲			1,187bp			1,181bp		
***ndhI*** **SSC**																								
																							
	506bp			506bp			506bp			514bp □			469bp □ ▲			520bp □			530bp			524bp		
***ndhJ*** **LSC**																								
																							
	476bp			476bp			476bp			434bp ■ ▲									476bp			476bp		
***ndhK*** **LSC**																								
																							
	701bp			677bp			677bp			410bp ▲									644bp			677bp		
	14,411bp (9.4%)			14,000bp (9.1%)			14,213bp (9.4%)			10,173bp (6.9%)			5,096bp (3.6%)			6,884bp (4.9%)			14,433bp (9.4%)			16,678bp (10.3%)		

^•^ Intron-containing gene

^▲^Missing fragment of coding region (incomplete gene)

^■^ Stop codon in-frame

* two gene copies

^□^ Frameshift

### Phylogenomic and phylogenetic analysis

#### The loss and recovery of the *ndh* gene complex among the Lentibulariaceae

**Fig 6:**
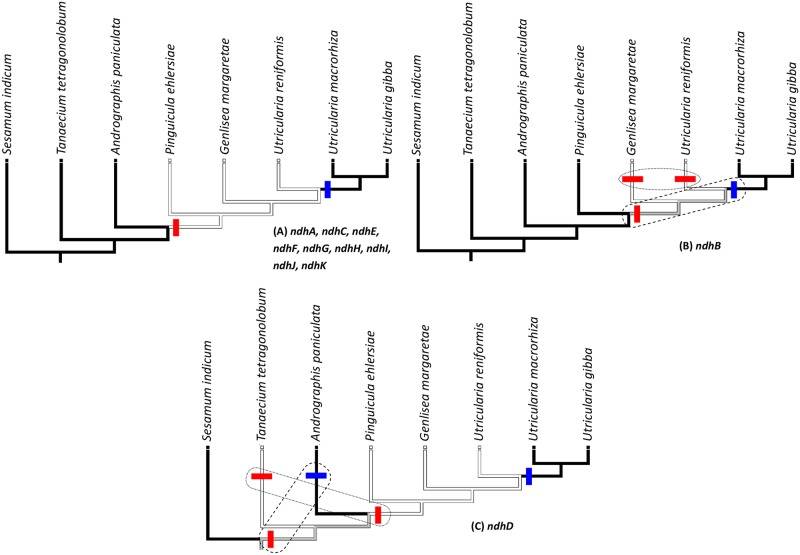
Phylogenetic history of *ndh* genes. **(A)** represents the only scenario for the *ndhA*, *ndhC*, *ndhE*, *ndhF*, *ndhG*, *ndhH*, *ndhI*, *ndhJ*, *and ndhK* genes evolution. **(B)** represents the phylogenetic history and the two possible scenarios for the *ndhB* gene and **(C)** for the *ndhD* gene (blue bars indicate the arising of the functional genes and red bars their loss).

Moreover, adding the *U*. *reniformis* plastome to more complete phylogenetic analyses, and tracing the possible evolutionary hypotheses according to the presence (functional gene) or absence (truncated or gene in frame-shift) of *ndh* genes, a more comprehensible evolutionary scenario can be determined. The phylogenetic history resulting from the cp phylogenomic analyses (for further discussion see below), indicates that Lentibulariaceae is a monophyletic family with the *Pinguicula* clade as a sister group of the *Genlisea-Utricularia* clade; the topology is also corroborated by previous studies [64,65]. By tracing the evolution of *ndh* genes in the trees, it was determined that the presence of functional *ndhA*, *ndhC*, *ndhE*, *ndhF*, *ndhG*, *ndhH*, *ndhI*, *ndhJ*, and *ndhK* is plesiomorphic, but were lost by pseudogenization or frame-shift in the ancestors of the Lentibulariaceae clade (Fig 6A). However, those *ndh* genes were recovered in a reversion process for the aquatic group, formed by *Utricularia gibba* and *U*. *macrorhiza*. A similar situation may have occurred with the *ndhB* gene, but this gene is functional for *Pinguicula* (Fig 6B). Even assuming the presence of this gene in the ancestral lineage, two possibilities can be explored by optimizing the transformations in the tree, and having both accepted with the parsimony approach (ACCTRAN and DELTRAN—[66]) since both hypotheses assume the same number of transformations (two in this case). Therefore, it is possible to accept the alternative scenario of the loss of *ndhB* gene from the ancestors of the *Genlisea-Utricularia* clade, or independent (parallel) losses for *Genlisea* and *Utricularia reniformis* (Fig 6B). For the same reasons, it is necessary to assume two alternative histories for the ndh*D* gene as well. The *ndhD* is functional for the aquatic species, *Utricularia gibba* and *U*. *macrorhiza*. It exists as a pseudogene for *Pinguicula* and *U*. *reniformis*, but as a frame-shift for *Genlisea* (Table 7). After surveying all Lamiales taxa represented in the phylogenomic analyses (Fig 6), we found that most species present a functional version of *ndhD* (*Tanaecium tetragonolobum* as an exception), thus it is reasonable to suppose this state as plesiomorphic. Assuming the topology as presented in Fig 6C, the *ndhD* gene can be lost independently more than once. In the present scenario, *ndhD* was lost for the *Tanaecium* and Lentibulariaceae clades, and afterwards reverted in the ancestors of the aquatic species *Utricularia gibba* and *U*. *macrorhiza* (Fig 6C), similar to the other *ndh* genes (Fig 6A and 6B). There is also the possibility that *ndhD* was lost in the ancestors of the *Tanaecium-Andrographis-Pinguicula-Genlisea-Utricularia* clade, and reverted twice for the *Andrographis* and *U*. *gibba—U*. *macrorhiza* clades. Both of these hypotheses for the *ndhD* gene assume three events, thus both are plausible.

#### Relationships with the Lamiales order

**Fig 7:**
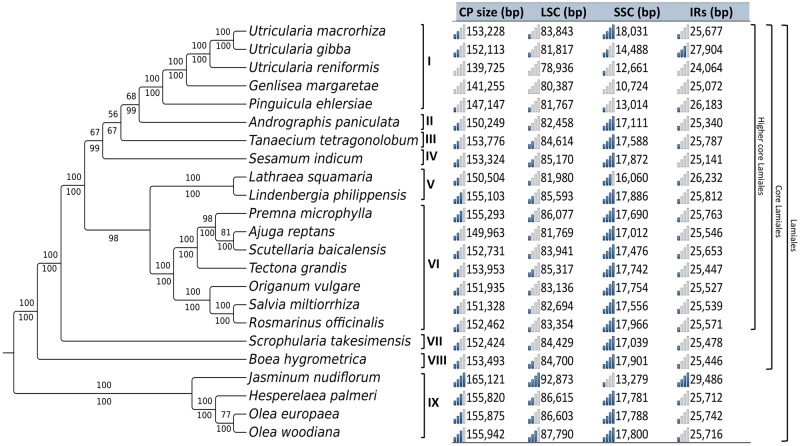
Phylogenomic analysis based on 23 complete Lamiales cp genomes. The phylogenetic position of *Utricularia reniformis* was inferred by maximum likelihood and Bayesian analyses. Numbers above and below the lines indicate the maximum likelihood bootstrap values and Bayesian posterior probabilities values > 50% for each clade, respectively. The table on the right indicates the genome features in base pairs (chloroplast genome length, LSC, SSC and IRs regions). The histograms located to the left of each feature (CP size, LSC, SSC and IRs), graphically illustrate the size distribution for each feature. Sub-titles: **I** Lentibulariaceae; **II** Acanthaceae; **III** Bignoniaceae; **IV** Pedaliaceae; **V** Orobanchaceae; **VI** Lamiaceae; **VII** Scrophulariaceae; **VIII** Gesneriaceae, and **IX** Oleaceae.

**Fig 8:**
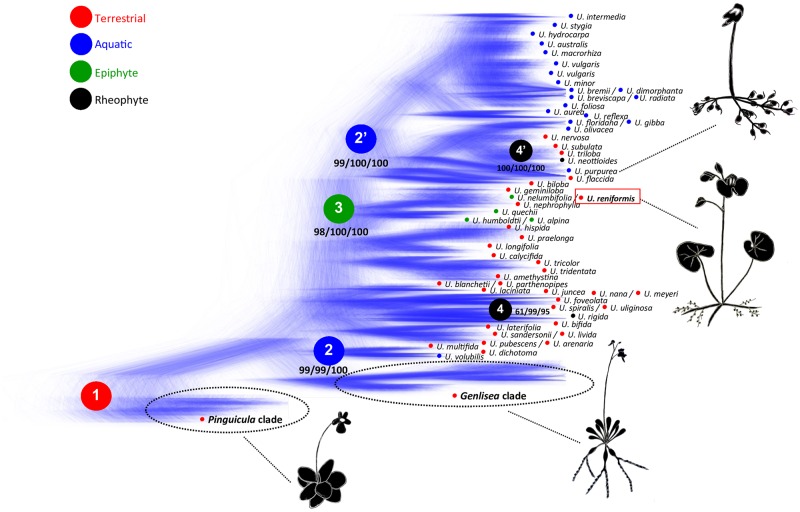
Cloudgram (Bayesian inference) of Lentibulariaceae from 18,000 Bayesian trees based on *matK* cp gene. The red circle 1 indicates the terrestrial ancestral lineage for the Lentibulariaceae family. The blue circles, 2 and 2’, represent the independent radiations to the aquatic habitat of *Utricularia* lineages. The green circle 3 indicates the possible ancestor of the epiphytic species from the plesiomorphic terrestrial life form, and the black circles, 4 and 4’, represent the independent origins for the rare reophytic life form for *Utricularia* lineages. Numbers below clades represent the support (maximum parsimony bootstrap/ maximum likelihood bootstrap/ posterior probabilities based on Bayesian inference). Higher color densities represent higher levels of certainty represented by congruent trees (from the 18,000 trees).

The relationships between the families in the order Lamiales are only partially resolved [67]. Despite the attempts based on several plastomes and mitochondrial genes [67,68] to identify the sister family of Lentibulariaceae, this issue still remains unclear. Therefore, phylogenomic analyses based on whole plastomes might contribute significantly to the elucidation of the systematic relationships inside of this order. With the aim to identify the phylogenetic position of *U*. *reniformis* and Lentibulariaceae among the other families of Lamiales, plastomes from 23 different taxa were compared. The phylogenomic analyses were constructed using 47 genes from the Lamiales plastomes. All Lamiales with less than 47 genes were excluded from our analysis, including parasitic plant cp genomes (truncated/pseudogenes and tRNAs were not considered). Both maximum likelihood (ML) and Bayesian analyses recovered the same tree topology with high support values (Fig 7), and agree with a previous study based on the rapidly evolving genes *trnK*/*matK*, *trn*L-F and *rps16* [68]. Our results indicate that Lentibulariaceae is monophyletic (100 maximum likelihood bootstrap—BML; 100 Bayesian posterior probability—PP) and *Pinguicula* is a sister clade of the *Genlisea-Utricularia* clade (100 BML; 100 PP), corroborating previous studies [65,69]. In addition, that *U*. *reniformis* is a sister to the *U*. *gibba*-*U*. *macrorhiza* clade, is also well supported (100 BML; 100 PP). According to our results based on *matK*, and also corroborated by previous studies [64,65], the ancestral lineage of Lentibulariaceae was possibly terrestrial, with life forms adapted for this environment developed by most species of *Pinguicula* and *Genlisea* (Fig 8). The alternative life forms present in *Utricularia* species [1,70] are thus derived from the ancestral terrestrial state, representing the occupation of different environments and the consequent diversification of body forms in an adaptive response to several ecological niches. Very specialized alternative life forms were developed by *Utricularia* lineages, for instance the rare reophytes, which inhabit rapids, cascades and streams at flood level during torrential conditions. For *Utricularia* this life form is represented only by the four species *U*. *neottioides*, *U*. *oliveriana*, *U*. *rigida*, and *U*. *tetraloba* [1], which have at least two independent phylogenetic origins (Fig 8, black circles 4 and 4’). Most aquatic species are represented by *Utricularia* sect. *Utricularia*(Taylor 1989), despite this life form having arisen at least twice (Fig 8, blue circles 2 and 2’), with species found in both the Northern and Southern Hemispheres (including *U*. *gibba* and *U*. *macrorhiza*). *Utricularia reniformis* is nested within a very specialized clade (Fig 8, green circle 3), with species adapted for terrestrial (the case of *U*. *reniformis*) and also epiphytic life forms, represented by species from sections *Iperua* and *Orchidioides*. The sister species of *U*. *reniformis* is *U*. *nelumbifolia* ([69]; this study), a rare endemic Brazilian *Utricularia*, very similar morphologically to *U*. *reniformis*, but which lives among the leaves of the giant bromeliads found on inselbergs [71]. These results also support positioning the Lentibulariaceae close to the Acanthaceae, Bignoniaceae, Pedaliaceae, Orobanchaceae, and Lamiaceae (Fig 7), composing the higher core of the Lamiales group previously proposed [68].

In general, the order Lamiales maintains the quadripartite structure, except for the parasitic family of Orobanchaceae that was not included in our analysis due to the small genome size (45kb to 120kb) and number of genes (21 to 42) [22]. In general the SSC and IR are 17 and 25kb long, respectively and only a few differences in genome size are observed (Fig 7). The biggest cp genome belongs to the family Oleaceae. The *Jasminum nudiflorum* cp genome is 165 kb with an IR and LSC expansion followed by a contraction in the SSC. This is also observed in *Schwalbea americana* from the Orobanchaceae family, which is 160kb long. In addition, SSC contraction and IR expansion were observed in the *Lathraea squamaria* from the Orobanchaceae family. However, major differences were noted in the Lentibulariaceae; SSC contraction was mainly observed in *Utricularia*-*Genlisea* and *Pinguicula* (Fig 7). This suggests that this family is under different selective pressures, resulting in dynamic plastome structures. It is noteworthy that only the Lentibulariaceae are carnivorous, suggesting their carnivorous syndrome may impact metabolism and photosynthesis.

## Discussion

### The *U*. *reniformis* plastome contribution to the study of the evolution of terrestrial carnivorous plants from the Lentibulariaceae family

We sequenced the cp genome of *Utricularia reniformis* and compared it against other carnivorous plants from the Lentibulariaceae family. This study revealed that the 139kbp cp genome of *U*. *reniformis* is quite similar to the cp genome of *U*. *gibba*, *U*. *macrorhiza*, *G*. *aurea*, and *P*. *ehlersiae* in terms of gene synteny, repeats and cpSSRs content; whereas the main differences are located on the SSC region and the *ndh* genes repertoire (Figs 3 and 5 and Tables 1, 2, 5 and 6). In spite of the similarity of the gene repertoire of the *U*. *reniformis* cp genome to essentially all photosynthetic organisms, comparative genomics analysis corroborated previous studies [15], which show that whereas aquatic forms maintain the complete *ndh* gene complex composed of eleven genes, the terrestrial forms have shown a number of losses of the *ndh* genes, and these losses are exclusive for each species (Table 7). In addition, the proposed phylogenetic history of *ndh* genes shown in Fig 6, suggests that independent *ndh* losses occurred during the course of the evolution of the genera; whether other terrestrial *Utricularia*, *Genlisea*, and *Pinguicula* species have also lost the *ndh* gene set, and whether the *ndh* pseudogenes found in the terrestrial forms were lost recently, remains to be investigated. Indeed, this is an important question to be explored in future work. Moreover, phylogenomic analysis supports that the family Lentibulariaceae is monophyletic, belonging to the higher core of the Lamiales clade, and thus corroborating the hypothesis that the first *Utricularia* lineage emerged in terrestrial habitats and then evolved to epiphytic and aquatic forms, as shown by the Fig 8.

Furthermore, the transcriptome analysis by RNAseq approach indicate that mostly cp genes are transcribed (Fig 2 and S3 Table), whereas even the truncated *ndh* genes, *orf42*, *orf56* and *ycf68* show some level of transcription. In addition to the previous observation that truncated transcribed molecules may exist in the chloroplast [56], this finding supports that the pervasive transcription, which is commonly found in bacterial genomes, may also occur in cp genomes, thus suggesting that these transcripts have an important role in gene regulation and genome evolution, as previous discussed elsewhere [72]. However, further studies are necessary to uncover the potential role of these transcripts. In addition, this study also shed some light on the RNA editing in cp genomes, with novel editing site being uncovered (Table 3), suggesting that the methodology used in this study represent a powerful tool to identify novel RNA editing sites.

### Endosymbiotic cp gene transfer to the nuclear and mitochondrial genome of *U*. *reniformis*

It was well known that during the course of evolution cp genes can be transferred to the nucleus, and their protein products can be reimported into the organelle lumen by the action of a transit peptide [20]. This indeed is a very widespread phenomenon in nature [73]. In addition, gene transfers from organelles often lead to functional replacement of host genes in a process called endosymbiotic gene replacement [21]. For instance, a large chunk of endosymbiotic cp genome was observed on chromosome 10 of rice, which contains a recent 33 kb insertion of cp DNA in addition to a 131 kb insertion representing nearly the entire plastid genome [74]. Furthermore, endosymbiotic gene transfer is also observed in smaller plant genomes, such as *A*. *thaliana* [75]. In our ongoing analysis of the *U*. *reniformis* genome we have noticed the presence of an endosymbiotic gene transfer of truncated cp genes to the nuclear and mitochondrial genomes. For instance, a total of 26kbp of cp-derived sequences were assembled in 28 contigs and mapped to mt and nuclear DNA assembled scaffolds (S2 Table). In addition to these contig-derived regions, during the ongoing assembly of the *U*. *reniformis* mt genome, we have found a truncated copy of *the*ndhJ, *ndhK* and *ndhC* genes (data not shown). Interestingly the *ndhJ*, *ndhK* and *ndhC* genes are absent from the *U*. *reniformis* cp genome (shown in details in the Fig 1 and Table 1). Indeed, this suggests that an ancient lineage of *U*. *reniformis* had these genes in their cp genome, which were subsequently transferred to the mt genome by an endosymbiotic event. However, due to evolutionary pressures, yet to be established, the *ndhJ*, *ndhK* and *ndhC* genes were decayed from the cp copies, and remained as relics in the mt genome. These observations also suggest that during the course of the evolution of the *ndh* complex in *U*. *reniformis*, endosymbiotic gene replacement events from the mt *ndhJ*, *ndhK* and *ndhC* copies, may have occurred. Further investigation is needed based on the sequencing of new species, and the presence and absence of the of *ndh* genes of others carnivorous plant cp and mt genomes. Therefore, the endosymbiotic gene transfer events are shaping the *U*. *reniformis* nuclear and mitochondrial genomes.

A detailed analysis of the *U*. *reniformis* nuclear and mt genomes, including functional annotation and comparative genomics showing the endosymbiotic transfer eventsbetween the organelles and the nuclear DNA are in progress.

### The impact of the carnivorous syndrome in the *ndh* complex genes evolution

It was previously observed that lineages that have lost photosynthetic function, trend toward reduced cp genome size [52]. The analysis of the cp genomes of the terrestrial forms, *Utricularia reniformis*, *Genlisea margaretae* and *Pinguicula ehlersiae*, may support this observation. However, *U*. *reniformis*, *G*. *margaretae* and *P*. *ehlersiae* are all photosynthetic organisms that lack the *ndh* genes, which apparently does not affect the fitness of these plants. Indeed, previous studies suggested that the *ndh* function might be dispensable under favorable growth conditions [19], suggesting that the carnivorous syndrome may act in favor of the functional *ndh* absence. Interestingly, the *ndh* gene loss or pseudogenization is relatively rare among the Viridiplantae clade [17,19]. However, it seems that the *ndh* genes were not essential during plant evolution, and their loss may also be related to early events leading to parasitic behavior [18]. In addition, the *ndh* genes are related to photosynthetic response to environmental stress, indicating its participation in the transition to terrestrial habitats [18,19]. However the first lineages of the Lentibulariaceae emerged in a terrestrial habitat, and then evolved to aquatic environments, suggesting that the evolutive history of the *ndh* genes among the Lentibulariaceae followed an opposite direction. For instance, we propose that the plastid *ndh* genes present in the aquatic forms *U*. *gibba* and *U*. *macrorhiza* were recovered in a reversion process, and that the *ndh* function may be dispensable in terrestrial forms (Fig 6).

In order to explain this genomic trend related to the loss of the *ndh* genes observed in the terrestrial Lentibulariaceae, a hypothesis posits that carnivorous plants are metabolically similar to parasitic plants in that they use organic carbon obtained through their prey, or their host for parasitic plants, to overcome environmental stress [15]. In addition, different and variable levels of nutritional stress to the plant may occur in aquatic, terrestrial, epiphytic and reophytic forms. This hypothesis is quite interesting, since it suggests that nutritional stress, which is a common feature of the carnivorous plants [2], can impact molecular and biochemistry characteristics shaping the cp genome. Moreover, over an evolutionary time scale these differences can lead to morphological changes, such as the adaptation to aquatic or land environments, and thus supporting the *ndh* gene repertoire differences observed among these species (Fig 8). However this hypothesis is yet to be established.

Conversely, it has been proposed that under relaxed selection, unequal efficiency of DNA repair, and high levels of mutagenic reactive oxygen species (ROS), the genome architecture of the Lentibulariaceae may also have been shaped in a fashion similar as those observed in the parasitic plants [9,14,15]. Indeed, a previous study has shown a genome-level convergence between carnivorous and parasitic plants [22]. Moreover, the *ndh* loci have accumulated several nucleotide substitutions and repeats [15], which may have resulted in the loss and pseudogenization process observed in *U*. *reniformis*, *G*. *margaretae* and *P*. *ehlersiae*. Indeed the sequence repeats are located mostly in the coding regions, and this is particularly noted with *ndhD*Ψ gene in *U*. *reniformis* (Table 6). However the sequencing of additional terrestrial and aquatic forms is necessary to corroborate the role of the sequence repeats with the pseudogenization process.

Overall, we propose that sequencing of additional cp and nuclear genomes from other individuals and species from the Lentibulariaceae family will shed light on the relationships between the rearrangement and loss of *ndh* genes, life style (aquatic, terrestrial, epiphytic and reophytic) and endosymbiotic gene transfer of cpDNA. Indeed, a recent study has shown that the endosymbiotic gene transfer has also occurred in other carnivorous plants, such as the *Genlisea nigrocaulis* and *G*. *hispidula* genomes [76], thus suggesting that this may be an evolutionary trend. Whether the *ndh* genes loss in terrestrial forms and the endosymbiotic gene transfer is an evolutionary trend of this group, which is leading to biochemistry and plastome-scale convergence with the parasitic plants, remains as an important question to be answered in the near future.

## Supporting Information

S1 Table Phylogenomic and phylogenetic analysis. **(A)** List of the chloroplast genomes used in the phylogenomics analysis with their respective GenBank accession number. **(B)** List of chloroplast genes used in the phylogenomics analysis. **(C)** The *matK* genes used in the phylogenetic analysis with their respective GenBank accession number. **(D)** The *matK* genes generated in this study and used in the phylogenetic analysis with their respective GenBank accession number.
(DOCX)

S2 Table The remaining paired-end reads (2x300bp; 229,830; 18.3%) which were assembled into contigs containing fragments of incomplete or truncated cp genes. The distribution, number of these contigs, truncated gene content and alignment to the *Utricularia reniformis* cp genome is shown.
(DOCX)

S3 Table RNAseq experiment table, showing the expression profile of all chloroplast related genes of *Utricularia reniformis*. (DOCX)
