# Supplementary material for: The Chloroplast Genome of Utricularia reniformis Sheds Light on the Evolution of the ndh Gene Complex of Terrestrial Carnivorous Plants from the Lentibulariaceae Family
Source: PLoS One. 2016 Oct 20;11(10):e0165176. doi: 10.1371/journal.pone.0165176 (PMC5072713; doi:10.1371/journal.pone.0165176)
Supplement: S1 Table — (A) List of the chloroplast genomes used in the phylogenomics analysis with their respective GenBank accession number. (B) List of chloroplast genes used in the phylogenomics analysis. (C) The matK genes used in the phylogenetic analysis with their respective GenBank accession number. (D) The matK genes generated in this study and used in the phylogenetic analysis with their respective GenBank accession number. (DOCX) [file pone.0165176.s001.docx]

**S1 Table.**

**A. Plastomes considered in the phylogenomics analysis**

| **Species** | **Family** | **Genbank acession #** |
| --- | --- | --- |
| *Andrographis paniculata* | Acanthaceae | KF150644 |
| *Tanaecium tetragonolobum* | Bignoniaceae | KR534325 |
| *Boea hygrometrica* | Gesneriaceae | JN107811 |
| *Ajuga reptans* | Lamiaceae | NC_023102 |
| *Origanum vulgare* | Lamiaceae | JX880022 |
| *Premna microphylla* | Lamiaceae | KM981744 |
| *Rosmarinus officinalis* | Lamiaceae | KR232566 |
| *Salvia miltiorrhiza* | Lamiaceae | JX312195 |
| *Scutellaria baicalensis* | Lamiaceae | KR233163 |
| *Tectona grandis* | Lamiaceae | HF567869 |
| *Genlisea margaretae* | Lentibulariaceae | NC_025652 |
| *Pinguicula ehlersiae* | Lentibulariaceae | NC_023463 |
| *Utricularia gibba* | Lentibulariaceae | NC_021449 |
| *Utricularia macrorhiza* | Lentibulariaceae | NC_025653 |
| *Hesperelaea palmeri* | Oleaceae | LN515489 |
| *Jasminum nudiflorum* | Oleaceae | DQ673255 |
| *Olea europaea* | Oleaceae | FN997650 |
| *Olea woodiana* | Oleaceae | FN998901 |
| *Lathraea squamaria* | Orobanchaceae | KM652488 |
| *Lindenbergia philippensis* | Orobanchaceae | HG530133 |
| *Sesamum indicum* | Pedaliaceae | NC_016433 |
| *Scrophularia takesimensis* | Scrophulariaceae | KM590983 |

**B. Chloroplast genes considered for the phylogenomic analysis**

*atpA, atpB, atpE, atpF, atpH, atpI, clpP, matK, petA, petG, petI, petN, psaA, psaB, psaC, psaI, psaJ, psbA, psbB, psbC, psbD, psbE, psbF, psbH, psbI, psbJ, psbK, psbL, psbM, psbN, psbT, rbcL, rpl2, rpl14, rpl20, rpl23, rpl36, rpoB, rpoC1, rps2, rps4, rps7, rps14, rps18, ycf2, ycf3, ycf4*

**C. *matK* accession numbers from GenBank Database used in the cloudgram analysis**

DQ010661, DQ010653, AF531782, AF531786, AF531814 ,FN641691. FN641690, FN641689, FN641695, FN641694, FN641714, FN641717, FN641716, FN641711, AF531838, AF531832, NC021449, AF531821, AF531840, AF531837, AF531851, AF531849, AF531828, AF531850, AF531830, FN773562, AF531827, JN894431, JN894028, AF531844, KC584950, AF531839, JN896195, AF531835, AF531831, AF531823, AF531847, AF531833, JN894029, JN894054, JN966728, JN894027, AF531846, AF531822, AF531836, AF531845, AF531825, AF531829, AF531843, AF531834, AF531824, AF531848, AF531826, AF531842, AF531841

**D. *matK* accession numbers generated in this study used in the cloudgram analysis**

KX604173, KX604174, KX604175, KX604176, KX604177, KX604178, KX604179, KX604180, KX604181, KX604182, KX604183, KX604184, KX604185, KX604186, KX604187, KX604188, KX604189, KX604190, KX604191, KX604192, KX604193, KX604194, KX604195, KX604196, KX604197, KX604198, KX604199, KX604200, KX604201, KX604202, KX604203, KX604204, KX604205, KX604206, KX604207, KX604208, KX604209, KX604210, KX604211, KX604212, KX604213, KX604214, KX604215, KX604216, KX604217, KX604218, KX604219, KX604220, KX604221, KX604222, KX604223, KX604224, KX604225, KX604226, KX604227, KX604228, KX604229, KX604230, KX604231
