# Supplementary material for: The Chloroplast Genome of Utricularia reniformis Sheds Light on the Evolution of the ndh Gene Complex of Terrestrial Carnivorous Plants from the Lentibulariaceae Family
Source: PLoS One. 2016 Oct 20;11(10):e0165176. doi: 10.1371/journal.pone.0165176 (PMC5072713; doi:10.1371/journal.pone.0165176)
Supplement: S2 Table — The distribution, number of these contigs, truncated gene content and alignment to the Utricularia reniformis cp genome is shown. (DOCX) [file pone.0165176.s002.docx]

**S2 Table.**
