# Supplementary material for: The Chloroplast Genome of Utricularia reniformis Sheds Light on the Evolution of the ndh Gene Complex of Terrestrial Carnivorous Plants from the Lentibulariaceae Family
Source: PLoS One. 2016 Oct 20;11(10):e0165176. doi: 10.1371/journal.pone.0165176 (PMC5072713; doi:10.1371/journal.pone.0165176)
Supplement: S3 Table — (DOCX) [file pone.0165176.s003.docx]

**S3 Table.**

| **Name** | **TPM** | **RPKM** | **Gene length** | **Unique gene reads** | **Total gene reads** |
| --- | --- | --- | --- | --- | --- |
| *accD* | 3630.39 | 5293.81 | 1473 | 886 | 886 |
| *atpA* | 18130.66 | 26437.99 | 1524 | 4578 | 4578 |
| *atpB* | 6898.44 | 10059.25 | 1497 | 1711 | 1711 |
| *atpE* | 3693.45 | 5385.76 | 402 | 246 | 246 |
| *atpF* | 9679.59 | 14114.71 | 588 | 943 | 943 |
| *atpH* | 30497.12 | 44470.66 | 246 | 1243 | 1243 |
| *atpI* | 3691.15 | 5382.4 | 744 | 455 | 455 |
| *ccsA* | 1910.97 | 2786.56 | 957 | 303 | 303 |
| *cemA* | 992.76 | 1447.64 | 687 | 113 | 113 |
| *clpP* | 13184.44 | 19225.44 | 591 | 1291 | 1291 |
| *infA* | 7789.58 | 11358.7 | 234 | 302 | 302 |
| *matK* | 2647.63 | 3860.75 | 1500 | 658 | 658 |
| *ndhA ψ* | 778.32 | 1134.94 | 1644 | 212 | 212 |
| *ndhB ψ* | 612.47 | 893.1 | 1084 | 110 | 110 |
| *ndhD ψ* | 924.16 | 1347.6 | 738 | 113 | 113 |
| *ndhE ψ* | 2218.22 | 3234.6 | 234 | 86 | 86 |
| *ndhG ψ* | 376.49 | 549 | 513 | 32 | 32 |
| *ndhH ψ* | 2084.13 | 3039.06 | 1086 | 375 | 375 |
| *ndhI ψ* | 275.91 | 402.34 | 525 | 24 | 24 |
| *orf42 ψ* | 33037.14 | 48174.51 | 114 | 624 | 624 |
| *orf56 ψ* | 11769.48 | 17162.17 | 120 | 234 | 234 |
| *petA* | 3653.97 | 5328.19 | 963 | 583 | 583 |
| *petB* | 18041.7 | 26308.26 | 648 | 1937 | 1937 |
| *petD* | 11034.09 | 16089.82 | 483 | 883 | 883 |
| *petG* | 52.94 | 77.2 | 114 | 1 | 1 |
| *petL* | 125.74 | 183.36 | 96 | 2 | 2 |
| *petN* | 1207.13 | 1760.22 | 90 | 18 | 18 |
| *psaA* | 7506.37 | 10945.72 | 2253 | 2802 | 2802 |
| *psaB* | 9024.71 | 13159.76 | 2205 | 3297 | 3297 |
| *psaC* | 22032.51 | 32127.64 | 246 | 898 | 898 |
| *psaI* | 18324.4 | 26720.49 | 111 | 337 | 337 |
| *psaJ* | 222021.85 | 323750.55 | 135 | 4966 | 4966 |
| *psbA* | 299974.91 | 437421.11 | 1059 | 52633 | 52633 |
| *psbB* | 19996.24 | 29158.37 | 1527 | 5059 | 5059 |
| *psbC* | 16120.49 | 23506.77 | 1422 | 3797 | 3798 |
| *psbD* | 7513.28 | 10955.81 | 1062 | 1322 | 1322 |
| *psbE* | 9197.15 | 13411.22 | 252 | 384 | 384 |
| *psbF* | 754.45 | 1100.14 | 120 | 15 | 15 |
| *psbH* | 7226.88 | 10538.17 | 228 | 273 | 273 |
| *psbI* | 598.13 | 872.18 | 111 | 11 | 11 |
| *psbJ* | 588.84 | 858.65 | 123 | 12 | 12 |
| *psbK* | 227.15 | 331.22 | 186 | 7 | 7 |
| *psbL* | 3043.61 | 4438.17 | 117 | 59 | 59 |
| *psbM* | 5460.81 | 7962.91 | 105 | 95 | 95 |
| *psbN* | 7910.34 | 11534.79 | 132 | 173 | 173 |
| *psbT* | 335.31 | 488.95 | 108 | 6 | 6 |
| *psbZ* | 1309.32 | 1909.24 | 189 | 41 | 41 |
| *rbcL* | 18439.4 | 26888.2 | 1434 | 4381 | 4381 |
| *rpl14* | 3467.63 | 5056.47 | 369 | 212 | 212 |
| *rpl16* | 5360.11 | 7816.07 | 411 | 365 | 365 |
| *rpl20* | 3472.11 | 5063.01 | 372 | 214 | 214 |
| *rpl22* | 4236.93 | 6178.26 | 453 | 318 | 318 |
| *rpl23* | 1005.94 | 1466.85 | 282 | 47 | 47 |
| *rpl2* | 16095.02 | 23469.63 | 828 | 2208 | 2208 |
| *rpl32* | 189.8 | 276.76 | 159 | 5 | 5 |
| *rpl33* | 6966.5 | 10158.5 | 201 | 232 | 232 |
| *rpl36* | 1535.38 | 2238.88 | 114 | 29 | 29 |
| *rpoA* | 8381.81 | 12222.29 | 993 | 1379 | 1379 |
| *rpoB* | 761.91 | 1111 | 3240 | 409 | 409 |
| *rpoC1* | 720.41 | 1050.5 | 2061 | 246 | 246 |
| *rpoC2* | 833.01 | 1214.69 | 4101 | 566 | 566 |
| *rps11* | 5181.67 | 7555.87 | 417 | 358 | 358 |
| *rps12* | 3504.56 | 5110.32 | 372 | 216 | 216 |
| *rps14* | 8246.71 | 12025.28 | 303 | 414 | 414 |
| *rps15* | 1786.05 | 2604.41 | 294 | 87 | 87 |
| *rps16* | 6080.84 | 8867.04 | 267 | 269 | 269 |
| *rps18* | 5883.79 | 8579.7 | 318 | 310 | 310 |
| *rps19* | 1514.32 | 2208.16 | 279 | 70 | 70 |
| *rps2* | 4903.95 | 7150.9 | 720 | 585 | 585 |
| *rps3* | 6758.8 | 9855.62 | 651 | 729 | 729 |
| *rps4* | 2539.75 | 3703.44 | 606 | 255 | 255 |
| *rps7* | 13244.86 | 19313.55 | 468 | 1027 | 1027 |
| *rps8* | 2399.35 | 3498.71 | 405 | 161 | 161 |
| *ycf1_small* | 40.37 | 58.87 | 897 | 2 | 6 |
| *ycf15* | 232.95 | 339.69 | 285 | 11 | 11 |
| *ycf1_large* | 3408.11 | 4969.67 | 4794 | 2314 | 2707 |
| *ycf2* | 509.17 | 742.46 | 6816 | 575 | 575 |
| *ycf3* | 7929.16 | 11562.25 | 510 | 670 | 670 |
| *ycf68 ψ* | 6264.83 | 9135.33 | 395 | 410 | 410 |
